# Preparation and Modeling of Graphene Bubbles to Obtain Strain-Induced Pseudomagnetic Fields

**DOI:** 10.3390/ma17122889

**Published:** 2024-06-13

**Authors:** Chuanli Yu, Jiacong Cao, Shuze Zhu, Zhaohe Dai

**Affiliations:** 1 Department of Mechanics and Engineering Science, State Key Laboratory for Turbulence and Complex Systems, College of Engineering, Peking University, Beijing 100871, China; yucl@stu.pku.edu.cn (C.Y.); cjc3037630482@stu.pku.edu.cn (J.C.); 2 Center for X-Mechanics, Department of Engineering Mechanics, Institute of Applied Mechanics, Zhejiang University, Hangzhou 310000, China; shuzezhu@zju.edu.cn

**Keywords:** pseudomagnetic field, graphene bubble, strain engineering, oxygen plasma, strain field

## Abstract

It has been both theoretically predicted and experimentally demonstrated that strain can effectively modulate the electronic states of graphene sheets through the creation of a pseudomagnetic field (PMF). Pressurizing graphene sheets into bubble-like structures has been considered a viable approach for the strain engineering of PMFs. However, the bubbling technique currently faces limitations such as long manufacturing time, low durability, and challenges in precise control over the size and shape of the pressurized bubble. Here, we propose a rapid bubbling method based on an oxygen plasma chemical reaction to achieve rapid induction of out-of-plane deflections and in-plane strains in graphene sheets. We introduce a numerical scheme capable of accurately resolving the strain field and resulting PMFs within the pressurized graphene bubbles, even in cases where the bubble shape deviates from perfect spherical symmetry. The results provide not only insights into the strain engineering of PMFs in graphene but also a platform that may facilitate the exploration of the strain-mediated electronic behaviors of a variety of other 2D materials.

## 1. Introduction

Two-dimensional (2D) materials have garnered significant attention owing to their unique structure—a 2D hexagonal lattice composed of carbon atoms—which imparts remarkable electronic properties to them [1,2,3,4,5]. In addition, the atomical thinness of 2D materials endows them with exceptional flexibility and mechanical deformability, further enabling the manipulation of their electronic structure through strain engineering methods [5,6,7,8,9,10]. For instance, it has been found that out-of-plane deformation can induce local changes in the electronic properties of graphene [9,10], leading to a shift in the electron’s Bloch wave vector, akin to the behavior observed in the presence of an external magnetic field [4,11,12]. This phenomenon has been termed strain-induced pseudomagnetic field (PMF) [4,11,13]. The intensity of such PMFs (which can be even greater than 300 Tesla) can surpass by several orders of magnitude that of the external magnetic field generated with a superconducting magnet, opening up exciting avenues for exploring novel quantum phenomena in graphene systems [4,14,15]. More broadly, strain engineering is a valuable tool for modifying the conductivity, magnetism, and various other electronic characteristics of 2D materials [16,17,18].

Strain engineering of traditional semiconductors typically encompasses inducing in-plane strains, for instance through lattice mismatch [19,20], thermal expansion mismatch [21,22], and deformation mismatch [23,24]. The extraordinary mechanical flexibility and deformability of 2D materials can give rise to new modes via out-of-plane deformations [6,16,17,25,26]. Examples include compression-induced wrinkles [27,28], conforming the atomic layer to patterned substrates [29,30,31,32], and direct application of transverse loads [8,16,33,34]. Among these, pressurizing the layer into bubble-like structures stands out as a viable means for the strain engineering of 2D materials [35,36,37,38]. The particular advantage of such a bubbling method lies in the relatively large strain magnitude (up to 5%) that it can achieve and the well-defined strain fields [35,39,40,41]. Recently, significant advancements have yielded a range of techniques for generating bubbles spontaneously or under external pressure [4,39,41,42,43,44,45,46,47,48,49,50,51,52,53,54,55,56,57,58,59,60,61,62,63,64,65,66,67,68,69,70,71,72,73,74,75,76,77,78,79,80,81,82,83,84,85,86,87,88,89,90,91,92]. Yet, leveraging 2D material bubbles for strain engineering (such as strain-induced PFM) typically demands cost-effectiveness, durability, and meticulous control over size and shape, presenting ongoing challenges.

Here, we first briefly discuss the advantages and disadvantages of typical bubbling methods in terms of durability and the controllability of the size and shape of the resulting bubbles. We then present a fast, simple bubbling technique relying on oxygen plasma chemical reactions. This method effectively demonstrates the rapid generation of out-of-plane deflections of graphene sheets. To accurately compute the strain field and resulting PMFs within the pressurized graphene bubbles, we develop a numerical scheme that is applicable to bubbles of any shape. The effect of the slippage of the graphene sheet over its supporting substrate on the PMFs is discussed. The experimental approach to inducing graphene bubbles and the computational method for analyzing the resulting strain fields can be applied to a variety of other 2D materials for the study of their fascinating strain-mediated electronic and optoelectronic behaviors.

## 2. A Brief Overview of Bubbling Methods

Nanobubbles can be created by inserting gas molecules between 2D materials and a substrate. Specifically, protons with specific energy can penetrate the 2D material and form gas molecules between layers or between layers and substrates, resulting in dome-shaped bubbles. For instance, Tedeschi et al. [42] and He et al. [43] reported that, via irradiating bulk crystals of transition metal dichalcogenides (TMDs) and hexagonal boron nitride (hBN) with low-energy hydrogen ions or hydrogen plasma, hydrogen ions could penetrate the uppermost layer to form gas-condensed bubbles filled with H_2_. Yasuda et al. utilized the permeability of graphene defect sites to hydrogen protons and the electrochemical hydrogen evolution reaction to construct hydrogen bubbles instead of using proton irradiation [44]. The height and radius of hydrogen proton-induced bubbles can be controlled within a range from a few nanometers to several hundred nanometers or even micrometers. In addition, the medium can also comprise other particles such as Ne^+^, Xe^+^, Ar^+^, and He^+^, but the resulting bubbles usually exhibit a height range that spans from subnanometer to several nanometers [45,46,47,48]. Controlling the height, shape, and position of the bubbles using these methods above has proven challenging [49]. To overcome this challenge, Polimeni and coworkers have recently achieved selective proton irradiation via creating a layer of H-opaque mask followed by proton irradiation, which led to a top array of single-layer controlled-size/position circles containing pressurized hydrogen [42,50,51].

In fact, in earlier stages of relevant strain engineering studies, PMFs from graphene bubbles were formed spontaneously via directly transferring the 2D material sheet onto a target substrate [4,39]. During the transfer process, gas, liquid, or foreign contaminants (such as residues of organic matter used for transfer) could become trapped on the surface of either the 2D material or the substrate [41,52,53,54,55,56,57,58,59,60,61,62]. After contact, these substances could become squeezed and aggregated at the interface due to the attractive van der Waals (vdW) interaction at the 2D material/substrate interface. It has also been reported that this type of bubble can be formed in the presence of defects within the material or at the material interface, through which gas and liquid molecules may migrate spontaneously to the layer/layer interface [63,64,65,66,67,68,69,70]. Recent attempts at the fabrication of 2D material bubbles have involved the intentional introduction of nanoparticles at the interface, giving rise to artificial means to control the process [71,72,73].

The density and size of such spontaneous bubbles tend to be random. In order to control the formation of bubbles, extensive efforts have employed external energy input to facilitate the intercalation of extra water or gas molecules into the interface of the 2D materials. For instance, an electron beam can induce the dissociation of water sandwiched between graphene layers to produce hydrogen gas, thereby triggering gas-filled graphene bubbles [74,75]. This also illustrates the potential of graphene as an effective chemical reactor and hydrogen storage container. Proton irradiation or chemical etching can also cause a chemical reaction in silicon dioxide substrate beneath the graphene, leading to bubble formation [76]. Applying a voltage allows the water within the interface to decompose into gas, resulting in bubbles with controllable size and shape [77,78]. Laser irradiation is another method that can activate specific chemicals. For example, chlorine trifluoride (ClF3) or liquid nitrogen (LN) embedded between graphite layers can induce bubbling under laser stimulation [79,80].

The construction of bubbles using 2D material can also be intentionally achieved through introducing liquid at the vdW interface [53,64,66,81,82,83]. Pantelis et al. demonstrated the construction of water-filled blisters at the MoS_2_/graphene interface through adjusting the air humidity, reaching heights of nearly 30 nm [81]. Although the introduction of liquid at the layer/substrate interface during wet transfer is unintentional, it provides a powerful platform to study the chemical and physical behavior of liquids in nanoscale confined spaces. Additionally, blisters can be manipulated through changing liquid composition and volume. Vasu et al. utilized this method to encapsulate salts or molecular solutions between graphene/graphene or graphene/graphite interfaces [84].

Methods utilizing pressure chambers to controllably prepare bubbles have increasingly been utilized for studying the physical, chemical, and mechanical properties of strained 2D materials. One such method was first reported by Bunch et al. [85]. Briefly, the 2D material was mechanically exfoliated or transferred onto a substrate with pre-patterned microcavities and then, the assembly was placed into a high-pressure chamber [85]. Within the chamber, gas molecules diffused along the interface between the material and the substrate, as well as through the pores of the substrate, until the pressure equalized (usually 4–6 days but highly dependent on the gas used) [35,38]; a pressure difference was thus created after taking out the sample, which induced gas-pressurized 2D material bubbles [86,87,88,89,90,91]. In addition, electrostatic force can be generated via applying a voltage, eliminating the need for a high-voltage chamber to drive the 2D material to bulge [92].

Table 1 presents a summary of the typical height, lateral size, controllability of shape and position, and durability of bubbles induced by the aforementioned methods. It should be noted that the table does not include all methods, due to unavailable data. Material thickness is disregarded, and durability is measured through the absence of a significant height decrease. The data rely solely on reported values, potentially underestimating the upper limits of corresponding methods. It is clear from Table 1 that there remain shortcomings such as lengthy bubble manufacturing cycles, complex device setups, and poor durability. These limitations hinder their utility in strain engineering applications as well as nanodevice applications including valleytronic devices, pressure sensors, and so on [2,93,94,95,96].

## 3. Experimental Method

**Figure 1:**
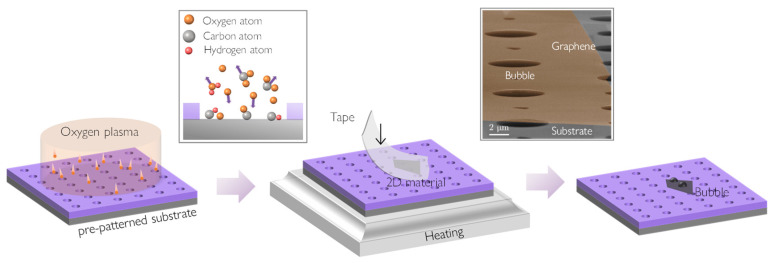
Schematic diagram of bubbles prepared via oxygen plasma-assisted method. The substrate with pre-patterned microcavities is pre-processed with oxygen plasma, after which the graphene is micro-mechanically exfoliated onto the treated substrate. Peeling the tape from the heated assembly can lead to the spontaneous bubbling of the transferred graphene sheet immediately at these pre-patterned cavities. The upper right panel shows an SEM image capturing graphene bubbles at a particular deflection angle.

Here, we report an alternative method that effectively combines the advantages of spontaneous bubbles and pressurized bubbles. Figure 1 shows a schematic diagram of the oxygen plasma-assisted preparation of graphene bubbles proposed in this work. Based on micro–nano processing technology, photolithography and reactive ion etching (RIE, ETCHLAB 200, SENTECH Inc., Berlin, Germany) were employed to fabricate an array of circular holes on the surface of a silicon wafer (with a silicon oxide layer thickness of 300 nm). Before use, the substrate with pre-patterned microcavities was subjected to pre-processing via oxygen plasma with a power of 100 W for 5 min. Note that oxygen plasma treatment (Plasma cleaner CPC-A, CIF International Group Co., Ltd, Compton, CA, USA) has previously been used to enhance the quality of ohmic contact via effectively cleaning the surface of 2D materials [62]. Next, the graphene was micro-mechanically exfoliated onto the treated substrate via Scotch tape (3M Co., Cottage Grove, MN, USA). Subsequently, the assembly was heated at 90 °C for 6 min to reduce the stickiness of the tape and increase the yield of the few-layer graphene. Note that this setting was flexible—we found heating at 90–110 °C for 4–7 min worked very well. Upon immediate removal of the tape after heating, graphite flakes of varying thicknesses remained on the substrate, and graphene bubbles of different heights and different layers were formed spontaneously. Finally, we used scanning electron microscopy (SEM, Quanta 600, FEI Inc., Hillsboro, OR, USA) to characterize the topology of the prepared samples. In the upper right image in Figure 1, bubbles can be seen forming on the holes covered by graphene. This observation was consistent across the few-layer graphene samples examined in other locations, confirming the effectiveness of our method and highlighting its advantages for mass production. Here, we demonstrate only three-layer graphene sheets, while emphasizing that this method should also be effective for other 2D materials with various numbers of layers.

The underlying mechanism is as follows. Pre-patterned substrates with microcavities invariably accumulate organic contaminations during micro/nanofabrication and subsequent storage [97,98]. Ultraviolet light produced in the plasma is highly effective in breaking down most organic bonds present in surface contaminants. This process aids in the breakdown of organic contaminations. At the same time, these organic compounds undergo vigorous oxidative chemical reactions when exposed to energetic oxygen species created in the plasma, as shown in Figure 1. Specifically, oxygen molecules undergo collision, dissociation, ionization, and recombination effects under the influence of high-speed electrons and the photoelectric effect, thereby generating oxygen plasma. Oxygen plasma comprises various particles, including $O_{2}^{+},O^{-},O_{2}^{-},O_{3}^{-}$, and excited-state energetic oxygen species such as $O_{2}^{*}$, among others. Notably, excited oxygen species can react with the organic layer on the substrate surface, resulting in the following chemical reactions: $organic+O_{2}^{*}\to {CO}_{2}+H_{2}O$ [99], leading to the spontaneous pressurization of the atomic sheet that seals these molecules into the cavity.

We note that oxygen plasma treatment is commonly employed for surface cleaning and substrate modification. However, due to the unique structure of the substrate, organic matter is adsorbed on both the substrate surface and the inner wall of cavities, albeit without uniformity. When oxygen plasma bombards the surface, the chemical reaction within the cavities persists longer than on the surface itself. This phenomenon arises from the relatively confined spatial characteristics of cavities, which concentrate chemical reactions. Furthermore, organic contaminations on the surface might experience physical removal due to the impact of high-energy particles, which is similar to the effect of sandblasting. In contrast, the depth within the microcavity limits the physical removal of contamination, thereby ensuring the chemical reactions occurring inside the cavity. Notably, although the primary purpose of heating is to remove adhesive and diminish tape–material adhesion, heating is also likely to make the chemical reaction in the cavity more complete, leading to increased gas filling into graphene bubbles.

## 4. Results and Discussion

### 4.1. Surface Topography

**Figure 2:**
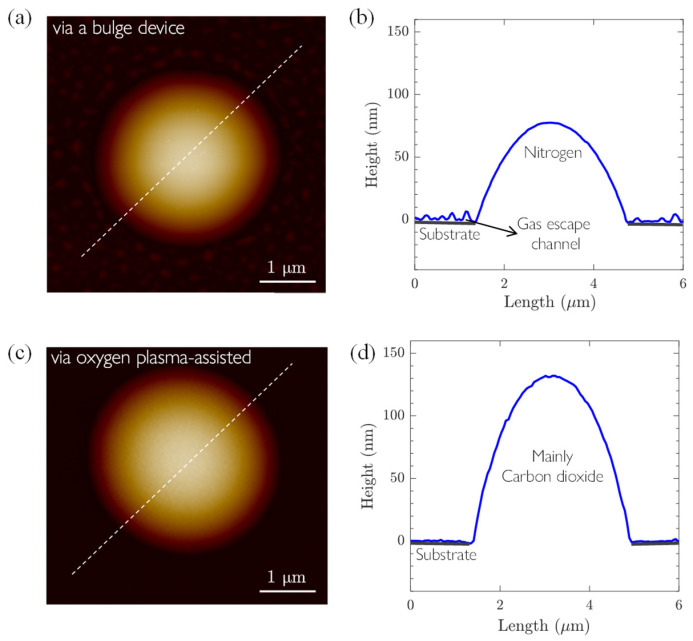
AFM height images of bubbles obtained via two preparation methods: (**a**,**b**) bubble prepared with a bulge device; (**c**,**d**) bubble prepared with the oxygen plasma-assisted method reported in this work. Note that both methods can lead to 1–3 layer graphene bubbles of heights ranging from 0 nm to 200 nm and a diameter of 3.5 μm.

Atomic force microscopy (AFM, Brucker Multimode 8) was employed to characterize the morphology of bubbles. The scanning was conducted in tapping mode with a scanning frequency of 0.7 Hz. An AFM cantilever featuring a silicon tip, with a resonance frequency of 150 kHz and a stiffness of 2.8 N/m, was utilized for the measurements. Figure 2a illustrates the height of bubbles fabricated via the oxygen plasma-assisted method, with a bubble height of about 130 nm. A comparative analysis with bubbles prepared using a bulge device revealed a conspicuous distinction. Particularly, bubbles prepared using a bulge device (similar to that in [85]) exhibited numerous minuscule bubbles at the graphene/substrate interface, as shown in Figure 2a,b. In contrast, these minuscule bubbles were absent in bubbles prepared using the oxygen plasma-assisted method. This difference confirmed the essential difference in the principles of the two bubbling methods. When using a bulge device, gas molecules are required to diffuse into the circular cavity through the channel between the material and the substrate. This diffusion causes gas molecules to accumulate at the interface, especially in the local contaminated areas, forming many nanobubbles and reducing interfacial adhesion. Furthermore, these smaller nanobubbles act as pathways for further gas diffusion, compromising the stability during the decay stage of the bubble. Conversely, the method proposed in this study effectively addresses this issue through directly generating gas from within the cavity, as shown in Figure 2d. Good interfacial integration should be able to slow down the escape of gas molecules. In addition, since bubble formation occurs through a rapid reaction, the preparation time of 2D materials bubbles is significantly reduced from over 4–6 days [13,35] to a few minutes. Therefore, this preparation method is high-throughput, with the number and size well controlled through the pre-patterning of the substrate, which can even be rectangular or other irregular shapes [37].

### 4.2. Deflating Behavior

It has been reported that even the smallest gas molecules (such as helium) and ions (such as lithium) cannot penetrate defect-free graphene [90]. At the graphene/SiO_2_ interface, however, bubbles prepared through gas pressurization via the bulge device can release most of the filled gas within 10 h for hydrogen-filled bubbles and within 7 days for nitrogen-filled bubbles [100,101]. In cases of strong interface adhesion, gas molecule diffusion is slow and mainly occurs through channels within the silicon dioxide. We then investigated the durability of bubbles prepared via the bugle device and the method reported in this work. We focused on four sets of bubble samples.

**Figure 3:**
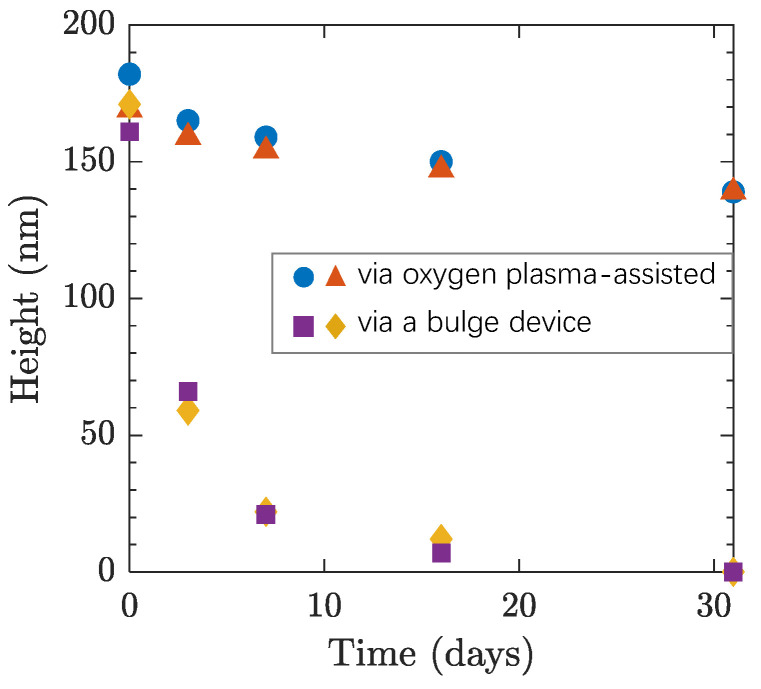
Height measurements of 3-layer thickness bubbles measured over 31 days. All bubbles showed signs of gradual deflation, indicating that the gas of the bubbles was able to escape through the graphene/SiO_2_ interface but at a much slower rate in bubbles prepared via the oxygen plasma-assisted method than the direct bulging method. For the same bubble, the height was measured along three different line scanning directions, and the error was found within 1 nm.

Over the subsequent 31 days, we conducted AFM scans on the same samples using consistent scanning parameters and a cantilever. Our results show that the height of the three-layer thickness graphene bubbles prepared using the oxygen plasma-assisted method decreased at different rates, but to a smaller extent, as shown in Figure 3. As a comparison, bubbles of the same thickness prepared using a bulge device experienced rapid height reduction, nearly flattening after 5 days. Based on the observation in Figure 2, it is reasonable to hypothesize that the difference attributed to the fast gas-release channel caused by adhesion imperfections at the graphene/SiO_2_ interface was largely suppressed in bubbles fabricated via our reported method.

## 5. Calculation of Strain and Pseudomagnetic Fields

We then moved on to assessment of the strain and potential pseudomagnetic fields in these prepared bubbles. Strain measurement within nanobubbles of moderate deflections can be achieved through utilizing approximate theories such as Föppl–von Kármán (FvK) equations. To solve such nonlinear partial differential equations, we followed the numerical scheme reported in [102], where a numerical approach based on the Chebyshev spectral method was used to solve the strain tensor of arbitrary symmetric 2D material bubbles. This method relied solely on morphological data obtained from AFM and the Poisson’s ratio of the 2D material. We made modifications to the boundary conditions in this numerical scheme to match the practical situation in typical 2D material bubbles (to be discussed shortly) and then calculate the strain components in the 2D material sheet:

(1) $$\epsilon_{xx}=\frac{\partial u_{x}}{\partial x}+\frac{1}{2}{\left(\frac{\partial h}{\partial x}\right)}^{2},\epsilon_{xy}=\frac{1}{2}\left(\frac{\partial u_{x}}{\partial y}+\frac{\partial u_{y}}{\partial x}+\frac{\partial h}{\partial x}\frac{\partial h}{\partial y}\right),\epsilon_{yy}=\frac{\partial u_{y}}{\partial y}+\frac{1}{2}{\left(\frac{\partial h}{\partial y}\right)}^{2},$$

where x and y are the 2D Cartesian coordinates, $u_{x}$ and $u_{y}$ are the in-plane displacements (to be solved), and h is the out-of-plane deflection of the sheet (that can be directly measured from the bubble).

Given the uncertainty regarding slip conditions at the graphene/SiO_2_ interface [39], we focused on two limiting cases (i.e., no-shear and no-slip conditions) to solve these strain components as well as the corresponding pseudomagnetic fields. In particular, an effective gauge field can be induced from the in-plane strains:

(2) $$A=A_{PMF}\left(\epsilon_{xx}-\epsilon_{yy},-2\epsilon_{xy}\right)$$

In the low-energy Dirac Hamiltonian, the coupling constant $A_{PMF}\approx 7\;\mu m\cdot T$ can be further linked to the hopping energy, Fermi velocity, and electron charge [103]. This gauge field induces shifts in the Dirac cones of graphene at points K and K’ in opposite directions, akin to the effect of a perpendicularly applied magnetic field. These PMFs can be associated with the strain-induced gauge field as follows:

(3) $$B_{PMF}=\nabla \times A$$

### 5.1. Numerical Scheme

Due to the significantly smaller thickness of the graphene sheet compared with the dimensions of the film and the moderate deflection of graphene sheets used in the production of typical bubbles, the material law remains linear and the classical large-deflection thin plate theory (i.e., FvK equations) can be applied to establish control equations for in-plane displacement, strain, and stress [104]. The equilibrium equation and constitutive equation can be written as:

(4) $$\frac{\partial \sigma_{xx}}{\partial x}+\frac{\partial \tau_{xy}}{\partial y}=0,\;\frac{\partial \sigma_{xy}}{\partial x}+\frac{\partial \tau_{yy}}{\partial y}=0$$

(5) $$\epsilon_{xx}=\frac{\sigma_{xx}-\nu \sigma_{yy}}{Y},\epsilon_{yy}=\frac{\sigma_{yy}-\nu \sigma_{xx}}{Y},\epsilon_{xy}=\frac{\tau_{xy}}{2\mu }$$

where Y is the in-plane Young’s modulus (i.e., Young’s modulus times thickness), ν is Poisson’s ratio, μ is the in-plane shear modulus. We used the Airy stress function χ to express stresses as follows:

(6) $$\sigma_{xx}=\frac{\partial^{2}\chi }{\partial y^{2}},\tau_{xy}=-\frac{\partial^{2}\chi }{\partial x\partial y},\sigma_{yy}=\frac{\partial^{2}\chi }{\partial y^{2}}$$

so that equilibrium Equation (4) was satisfied automatically. Since the in-plane strain needs to satisfy the coordination relationship, the strain is expressed in the form of a stress function and brought into the coordination equation to obtain the compatibility equation of the FvK equations in the following form:

(7) $$\nabla^{2}\nabla^{2}\chi +Y\left\{\frac{\partial^{2}h}{\partial x^{2}}\frac{\partial^{2}h}{\partial y^{2}}-{\left(\frac{\partial^{2}h}{\partial x\partial y}\right)}^{2}\right\}=0$$

The above equation is equivalent to the following two coupled equations:

(8) $$\nabla^{2}\phi =-Y\left\{\frac{\partial^{2}h}{\partial x^{2}}\frac{\partial^{2}h}{\partial y^{2}}-{\left(\frac{\partial^{2}h}{\partial x\partial y}\right)}^{2}\right\}$$

(9) $$\nabla^{2}\chi =\phi$$

**Figure 4:**
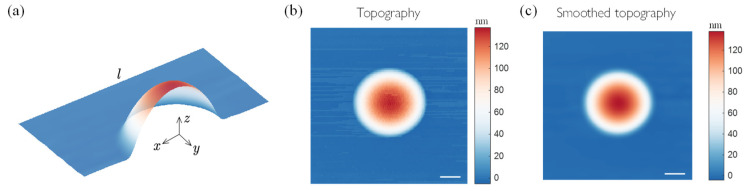
(**a**) Schematic diagram of the bubble model: the topography of bubble (**b**) before smoothing and (**c**) after smoothing. Scale bars: 1 μm.

Following our experiments, we considered a square region characterized by a central nanobubble with a length l as the computational region, as illustrated in Figure 4a. We assumed that the film was flat within a small neighborhood at the boundaries of the square region, i.e., at these boundaries, the partial derivatives of the height along the x and y directions were zero. Since the boundary was far enough away from the center of the bubble, we assumed that the films in the neighborhood were under the same simple stress state. At the same time, we assumed that the tension applied at the film boundary was constant. Therefore, in the boundary neighborhood, the stress tensor of the film can be expressed as:

(10) $$\underline{\underline{\sigma }}=\left[\begin{array}{cc} T_{xx} & T_{xy}\\ T_{xy} & T_{yy} \end{array}\right]$$

where $T_{xx}$, $T_{yy}$, and $T_{xy}$ represent the constant normal and shear stresses applied in the x and y directions, respectively. We note that the far-field stresses were set to be zero in [102].

At the boundary, the relationship between stress components and the stress function χ is established as:

(11) $$\partial^{2}\chi /\partial y^{2}=T_{xx},\;\;\partial^{2}\chi /\partial x^{2}=T_{yy},\;\;\partial^{2}\chi /\partial x\partial y=-T_{xy}.$$

This leads to the expression for ϕ, the Laplacian of χ:

(12) $$\phi =\nabla^{2}\chi =T_{xx}+T_{yy}$$

Considering these nontrivial boundary conditions, we integrate the stress components to derive $A\left(x,y\right)$ and $B\left(x,y\right)$:

(13) $$A\left(x,y\right)=\int_{{(x}_{0},y_{0})}^{\left(x,y\right)}-\tau_{xy}\left(\xi ,\eta \right)d\xi +\sigma_{xx}\left(\xi ,\eta \right)d\eta$$

(14) $$B\left(x,y\right)=\int_{{(x}_{0},y_{0})}^{\left(x,y\right)}-\sigma_{y}\left(\xi ,\eta \right)d\xi +\tau_{xy}\left(\xi ,\eta \right)d\eta$$

Using $A\left(x,y\right)$ and $B\left(x,y\right)$, we find $\chi \left(x,y\right)$, where ${\chi_{0}=c}_{1}x+c_{2}y+c_{3}$ represents the Laplace equation solution:

(15) $$\chi \left(x,y\right)=\frac{1}{2}T_{xx}y^{2}+\frac{1}{2}T_{yy}x^{2}-T_{xy}xy+c_{1}x+c_{2}y+c_{3},$$

where $c_{1}$, $c_{2}$, and $c_{3}$ are integration constants. For the stress/strain field distribution, we focused on the second-order partial derivatives of χ, hence ignoring the second-order terms. Thus, at the boundary, we simplify $\chi \left(x,y\right)$ to:

(16) $$\chi \left(x,y\right)=\frac{1}{2}T_{xx}y^{2}+\frac{1}{2}T_{yy}x^{2}-T_{xy}xy$$

The height information of a nanofilm can be extracted from AFM scanning images. However, these images often contain noise, resulting in an irregular height function with numerous artifacts. To address this, Gaussian filtering was applied to the interpolated data obtained from the original AFM scans. As shown in Figure 4b,c, this filtering process effectively removed extraneous noise, resulting in a smoother image and more accurate calculation results.

Following [102], we applied the Chebyshev spectral method to solve the FvK equations. This method relies on Chebyshev polynomials as basis functions, enabling accurate approximation of functions with fewer grid points compared to other numerical methods. The Chebyshev spectral method represents functions as finite series expansions of smooth basis functions. In this approach, differentiation operations are transformed into algebraic operations, simplifying the solution process. Taking the [−1,1]×[−1,1] interval as an example, with Chebyshev points defined as:

(17) $$x_{i}=\cos\left(i\pi /N\right),\;i=0,1,2,\ldots ,N\;andy_{j}=\cos\left(j\pi /N\right),\;j=0,1,2,\ldots ,N$$

for convenience, we may rewrite the Laplacian operator as:

(18) $${L=D}_{N}^{2}⊗I+I⊗D_{N}^{2}$$

where $D_{N}$ is an $\left(N+1\right)\times (N+1)$ matrix, and ⊗ denotes the Kronecker product. As a result, the FvK equations can be rewritten as:

(19) Lϕ=f  and  Lχ=ϕ,

where $f\left(x,y\right)=-Y\left\{\frac{\partial^{2}h}{\partial x^{2}}\frac{\partial^{2}h}{\partial y^{2}}-{\left(\frac{\partial^{2}h}{\partial x\partial y}\right)}^{2}\right\}$ can be measured from the height image of the bubble. To incorporate boundary conditions, the original matrices are rescaled based on experimental dimensions, resulting in equations satisfying the boundary conditions:

(20) $$\hat{L}\phi =\hat{f}\;\;and\;\;\;\hat{L}\chi =\hat{\phi }$$

These equations are then solved through successively taking the inverse of the modified matrices. It should be noted here that this numerical scheme is limited to 2D materials of FvK-type behavior and smooth distributions on strains (i.e., it is not capable of describing detailed dislocation stress and pinning stress at the material–material interface).

### 5.2. Numerical Results

**Figure 5:**
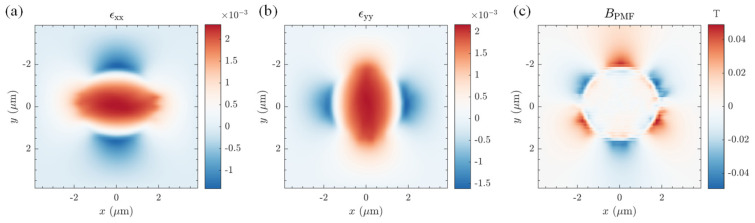
Numerical results of strain fields (**a**,**b**) and PMFs (**c**) induced through graphene bubbles prepared via the oxygen plasma-assisted method under no-shear conditions. The geometry of the bubble in this demonstration is described in Figure 4.

For a case where there is no shear between the film and the substrate, and the graphene–substrate interface is completely slippery, the boundary condition at the far field can be simplified to the film boundary stress being free with no tension. The resulting strain field and PMFs are shown in Figure 5. Figure 5a,b illustrates that the stress distribution induced via bubbling in graphene decreased with increasing distance from the center and exhibited larger strain gradients near the edge of the bubble. Such non-uniform lattice distortion can alter the low-energy electronic band structure of graphene. The magnitude of the electron transition between carbon atoms mimics the impact of an actual magnetic field perpendicular to the graphene plane. As depicted in Figure 5c, the PFMs generated by the axially symmetric graphene bubble exhibited three-fold symmetry, being most pronounced near the bubble’s periphery and attenuating rapidly towards its center. This result agrees with previous calculations using simplified theories or different methods, in both distribution and magnitude [105,106]. We also found that the maximum magnitude of PFM created this way was proportional to the square of the height-to-radius ratio of the graphene bubble and inversely proportional to the radius of the bubble. In this sense, the potential exists to achieve large PFMs in future studies through preparing bubbles of small radii and large heights using the oxygen plasma-assisted method we report.

We have discussed a limiting case of zero shear resistance at the graphene–substrate interface. In cases where the substrate underneath, such as silicon oxide, can provide a pinning effect, no-slip conditions would appear more appropriate [39,107]. However, a complex boundary layer arises from geometric irregularities of the bubble footprint. Hence, we designated the bubble boundary at a height of 1 nm as its cut-off bottom boundary. As the displacement of the bubble boundary is zero at no-slip conditions, we approximated the boundary condition via applying a biaxial in-plane pulling force at the far field membrane boundary, ensuring zero displacements of the bubble boundary. This approach rendered the governing equations and boundary conditions identical inside the bubble boundary equivalent to the no-slip situation. However, as the boundary was fixed, we disregarded the region outside the bubble boundary. It is important to note that although the geometric equation is nonlinear, the nonlinearity solely appears in the out-of-plane height term, which can be accurately determined through measurement.

Specifically, we began by fitting the contour of the axisymmetric nanobubble using the polynomial function:

(21) $$w\left(r\right)=a_{1}\left(1-{\left(\frac{r}{a}\right)}^{2}\right)+a_{2}\left(1-{\left(\frac{r}{a}\right)}^{4}\right)+a_{3}\left(1-{\left(\frac{r}{a}\right)}^{6}\right),\;\;r<a$$

**Figure 6:**
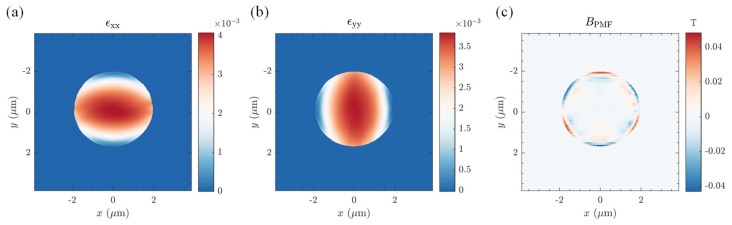
Numerical results of strain fields (**a**,**b**) and PMFs (**c**) induced through graphene bubbles prepared via the oxygen plasma-assisted method under no-slip conditions. The geometry of the bubble in this demonstration is described in Figure 4.

A constant tension T is applied at infinity to enforce a zero displacement at the boundary r=a. This requires:

(22) $$T=\frac{\left(15a_{1}^{2}+30a_{2}^{2}+72a_{2}a_{3}+45a_{3}^{2}+5a_{1}\left(8a_{2}+9a_{3}\right)\right)Y\left(1+\nu \right)}{60a^{2}\left(1-\nu \right)}$$

according to the analytical calculation of the axisymmetric version of Equation (19). Using w(r) to fitted the *x* and *y* directions of the bubble respectively, we obtained the corresponding parameters $a_{1x},\;a_{2x},\;a_{3x}$ and $a_{1y},\;a_{2y},\;a_{3y}$. These parameters were then substituted into the Equation (22) to derive $T_{x}$ and $T_{y}$. We approximated that through applying $T_{x}$ and $T_{y}$, respectively, at the membrane boundary to constrain the displacement of the nanobubbles at the boundary to zero. Subsequently, the strain field within the boundaries was obtained, as shown in Figure 6a,b.

Consequently, the solution involving constant tension at the membrane boundaries was equivalent to superposing the solution with no-shear conditions and the solution for a membrane boundary subjected to constant tensions. The strain field of the latter remained constant with a strain gradient of zero, thus not contributing to the PMFs. Furthermore, since we did not consider the strain distribution outside the bubble boundary, it was set to zero. The resulting PMF is shown in Figure 6c. It can be seen that the same bubble under no-slip conditions (Figure 6a,b) can provide large strain magnitudes compared to that under no-shear conditions (Figure 5a,b). Again, we found good agreement in both distribution and magnitude between the results in Figure 6 and previous calculations using simplified theories and clamped boundary conditions [105,106], while the numerical scheme discussed here could be applied to the solution of PMF of bubbles of arbitrary shapes.

## 6. Conclusions

In this study, we propose a rapid bubbling method based on chemical reactions between oxygen plasma and organic matter, enabling the rapid induction of in-plane strain and out-of-plane deformation in graphene and other 2D materials. Compared with other bubbling preparation methods, this approach has the advantages of short preparation time, simple operation, controllable bubble shape and position, and good durability. Furthermore, we introduce a numerical method to calculate the strain of the bubble and its induced three-fold symmetric pseudomagnetic field under both no-shear and no-slip conditions. This method has broad significance for studying the evolution behavior of electronic structures in strain-induced two-dimensional materials and for the development and application of valleyronic devices and micro–nano sensors.

## Figures and Tables

**Table 1:** Summary of typical characteristics of bubbles prepared via different methods.

Methods	Reference	Max. Height (nm)	Max. Lateral Size (nm)	Shape	Position	Durability
Proton irradiation-assisted	[42]	~500	~3000	Circular	Controlled	>2 years
	[43]	~200	~9600	Near-circular	Uncontrolled	>40 weeks
	[46]	~0.3	~12	Irregular	Uncontrolled	NA (Not applicable)
	[48]	267	4540	Near-circular	Uncontrolled	>23 months
	[49]	482	6300	Circular	Uncontrolled	NA
	[50]	422	5140	Circular	Uncontrolled	>2 years
Spontaneous formation	[67]	~60	~1000	Irregular	Uncontrolled	NA
	[70]	~100	Micron	Irregular	Uncontrolled	NA
Water splitting	[77]	~100	~2000	Nearly circular	Uncontrolled	NA
Hydrogen desorption	[78]	25	~400	Nearly circular	Controlled	NA
Laser irradiation	[79]	325	5600	Nearly circular	Controlled	NA
	[80]	~3.6	~68	Nearly circular	Uncontrolled	<12 days
Liquid introduction	[81]	~130	~450	Irregular	Uncontrolled	NA
	[82]	~850	~30,000	Irregular	Uncontrolled	>58 days
Direct bulging	[37]	~130	9000	Rectangular	Controlled	NA
	[85]	175	4750	Square	Controlled	<10 h
	[86]	~580	~7300	Circular	Controlled	>20 min

## Data Availability

The original contributions presented in the study are included in the article, further inquiries can be directed to the corresponding author.

## References

[1] Novoselov K.S., Geim A.K., Morozov S.V., Jiang D., Zhang Y., Dubonos S.V., Grigorieva I.V., Firsov A.A. (2004). Electric field effect in atomically thin carbon films. Science.

[2] Schaibley J.R., Yu H., Clark G., Rivera P., Ross J.S., Seyler K.L., Yao W., Xu X. (2016). Valleytronics in 2D materials. Nat. Rev. Mater..

[3] Hu H., Chen N., Teng H., Yu R., Xue M., Chen K., Xiao Y., Qu Y., Hu D., Chen J. (2023). Gate-tunable negative refraction of mid-infrared polaritons. Science.

[4] Levy N., Burke S.A., Meaker K.L., Panlasigui M., Zettl A., Guinea F., Neto A.H.C., Crommie M.F. (2010). Strain-Induced Pseudo–Magnetic Fields Greater Than 300 Tesla in Graphene Nanobubbles. Science.

[5] Androulidakis C., Koukaras E.N., Paterakis G., Trakakis G., Galiotis C. (2020). Tunable macroscale structural superlubricity in two-layer graphene via strain engineering. Nat. Commun..

[6] Si C., Sun Z., Liu F. (2016). Strain engineering of graphene: A review. Nanoscale.

[7] Wang B., Li J., Fang Z., Jiang Y., Li S., Zhan F., Dai Z., Chen Q., Wei X. (2024). Large and Pressure-Dependent *c*-Axis Piezoresistivity of Highly Oriented Pyrolytic Graphite near Zero Pressure. Nano Lett..

[8] Fang Z., Dai Z., Wang B., Tian Z., Yu C., Chen Q., Wei X. (2023). Pull-to-Peel of Two-Dimensional Materials for the Simultaneous Determination of Elasticity and Adhesion. Nano Lett..

[9] Stegmann T., Szpak N. (2016). Current flow paths in deformed graphene: From quantum transport to classical trajectories in curved space. New J. Phys..

[10] Stegmann T., Szpak N. (2019). Current splitting and valley polarization in elastically deformed graphene. 2D Mater..

[11] Low T., Guinea F. (2010). Strain-Induced Pseudomagnetic Field for Novel Graphene Electronics. Nano Lett..

[12] Rechtsman M.C., Zeuner J.M., Tünnermann A., Nolte S., Segev M., Szameit A. (2013). Strain-induced pseudomagnetic field and photonic Landau levels in dielectric structures. Nat. Photon..

[13] Zhang D.-B., Seifert G., Chang K. (2014). Strain-Induced Pseudomagnetic Fields in Twisted Graphene Nanoribbons. Phys. Rev. Lett..

[14] Settnes M., Power S.R., Brandbyge M., Jauho A.-P. (2016). Graphene Nanobubbles as Valley Filters and Beam Splitters. Phys. Rev. Lett..

[15] Zhu S., Huang Y., Klimov N.N., Newell D.B., Zhitenev N.B., Stroscio J.A., Solares S.D., Li T. (2014). Pseudomagnetic fields in a locally strained graphene drumhead. Phys. Rev. B.

[16] Dai Z., Liu L., Zhang Z. (2019). Strain Engineering of 2D Materials: Issues and Opportunities at the Interface. Adv. Mater..

[17] Deng S., Sumant A.V., Berry V. (2018). Strain engineering in two-dimensional nanomaterials beyond graphene. Nano Today.

[18] Qi Y., Sadi M.A., Hu D., Zheng M., Wu Z., Jiang Y., Chen Y.P. (2023). Recent Progress in Strain Engineering on Van der Waals 2D Materials: Tunable Electrical, Electrochemical, Magnetic, and Optical Properties. Adv. Mater..

[19] Wang Y., Yang R., Shi Z., Zhang L., Shi D., Wang E., Zhang G. (2011). Super-Elastic Graphene Ripples for Flexible Strain Sensors. ACS Nano.

[20] Zhao Q., Frisenda R., Wang T., Castellanos-Gomez A. (2019). InSe: A two-dimensional semiconductor with superior flexibility. Nanoscale.

[21] Islam A., Akker A.v.D., Feng P.X.-L. (2018). Anisotropic Thermal Conductivity of Suspended Black Phosphorus Probed by Opto-Thermomechanical Resonance Spectromicroscopy. Nano Lett..

[22] Wang F., Zhou B., Sun H., Cui A., Jiang T., Xu L., Jiang K., Shang L., Hu Z., Chu J. (2018). Difference analysis model for the mismatch effect and substrate-induced lattice deformation in atomically thin materials. Phys. Rev. B.

[23] Mohiuddin T.M.G., Lombardo A., Nair R.R., Bonetti A., Savini G., Jalil R., Bonini N., Basko D.M., Galiotis C., Marzari N. (2009). Uniaxial strain in graphene by Raman spectroscopy:Gpeak splitting, Grüneisen parameters, and sample orientation. Phys. Rev. B.

[24] Raju A.P.A., Lewis A., Derby B., Young R.J., Kinloch I.A., Zan R., Novoselov K.S. (2014). Wide-Area Strain Sensors based upon Graphene-Polymer Composite Coatings Probed by Raman Spectroscopy. Adv. Funct. Mater..

[25] Hou Y., Zhou J., Xue M., Yu M., Han Y., Zhang Z., Lu Y. (2024). Strain Engineering of Twisted Bilayer Graphene: The Rise of Strain-Twistronics. Small.

[26] Liu S., He J., Rao Y., Dai Z., Ye H., Tanir J.C., Li Y., Lu N. (2023). Conformability of flexible sheets on spherical surfaces. Sci. Adv..

[27] Castellanos-Gomez A., Roldán R., Cappelluti E., Buscema M., Guinea F., van der Zant H.S.J., Steele G.A. (2013). Local Strain Engineering in Atomically Thin MoS_2_. Nano Lett..

[28] Quereda J., San-Jose P., Parente V., Vaquero-Garzon L., Molina-Mendoza A.J., Agraït N., Rubio-Bollinger G., Guinea F., Roldán R., Castellanos-Gomez A. (2016). Strong Modulation of Optical Properties in Black Phosphorus through Strain-Engineered Rippling. Nano Lett..

[29] Martella C., Mennucci C., Cinquanta E., Lamperti A., Cappelluti E., de Mongeot F.B., Molle A. (2017). Anisotropic MoS_2_ Nanosheets Grown on Self-Organized Nanopatterned Substrates. Adv. Mater..

[30] Mangu V.S., Zamiri M., Brueck S.R.J., Cavallo F. (2017). Strain engineering, efficient excitonic photoluminescence, and exciton funnelling in unmodified MoS_2_ nanosheets. Nanoscale.

[31] Chen Z., Leng K., Zhao X., Malkhandi S., Tang W., Tian B., Dong L., Zheng L., Lin M., Yeo B.S. (2017). Interface confined hydrogen evolution reaction in zero valent metal nanoparticles-intercalated molybdenum disulfide. Nat. Commun..

[32] Zhang Y., Heiranian M., Janicek B., Budrikis Z., Zapperi S., Huang P.Y., Johnson H.T., Aluru N.R., Lyding J.W., Mason N. (2018). Strain Modulation of Graphene by Nanoscale Substrate Curvatures: A Molecular View. Nano Lett..

[33] Yang S., Chen Y., Jiang C. (2021). Strain engineering of two-dimensional materials: Methods, properties, and applications. InfoMat.

[34] Chen E., Dai Z. (2023). Axisymmetric Peeling of Thin Elastic Films: A Perturbation Solution. J. Appl. Mech..

[35] Cui X., Liu L., Dong W., Zhou Y., Zhang Z. (2023). Mechanics of 2D material bubbles. Nano Res..

[36] Darlington T.P., Carmesin C., Florian M., Yanev E., Ajayi O., Ardelean J., Rhodes D.A., Ghiotto A., Krayev A., Watanabe K. (2020). Imaging strain-localized excitons in nanoscale bubbles of monolayer WSe2 at room temperature. Nat. Nanotechnol..

[37] Cui X., Dong W., Feng S., Wang G., Wang C., Wang S., Zhou Y., Qiu X., Liu L., Xu Z. (2023). Extra-High Mechanical and Phononic Anisotropy in Black Phosphorus Blisters. Small.

[38] Sanchez D.A., Dai Z., Lu N. (2021). 2D Material Bubbles: Fabrication, Characterization, and Applications. Trends Chem..

[39] Dai Z., Lu N., Liechti K.M., Huang R. (2020). Mechanics at the interfaces of 2D materials: Challenges and opportunities. Curr. Opin. Solid State Mater. Sci..

[40] Wang G., Dai Z., Xiao J., Feng S., Weng C., Liu L., Xu Z., Huang R., Zhang Z. (2019). Bending of Multilayer van der Waals Materials. Phys. Rev. Lett..

[41] Chen Y., Wang Y., Shen W., Wu M., Li B., Zhang Q., Liu S., Hu C., Yang S., Gao Y. (2022). Strain and Interference Synergistically Modulated Optical and Electrical Properties in ReS_2_/Graphene Heterojunction Bubbles. ACS Nano.

[42] Tedeschi D., Blundo E., Felici M., Pettinari G., Liu B., Yildrim T., Petroni E., Zhang C., Zhu Y., Sennato S. (2019). Controlled Micro/Nanodome Formation in Proton-Irradiated Bulk Transition-Metal Dichalcogenides. Adv. Mater..

[43] He L., Wang H., Chen L., Wang X., Xie H., Jiang C., Li C., Elibol K., Meyer J., Watanabe K. (2019). Isolating hydrogen in hexagonal boron nitride bubbles by a plasma treatment. Nat. Commun..

[44] Yasuda S., Tamura K., Terasawa T.-O., Yano M., Nakajima H., Morimoto T., Okazaki T., Agari R., Takahashi Y., Kato M. (2020). Confinement of Hydrogen Molecules at Graphene-Metal Interface by Electrochemical Hydrogen Evolution Reaction. J. Phys. Chem. C.

[45] Wang J., Sorescu D.C., Jeon S., Belianinov A., Kalinin S.V., Baddorf A.P., Maksymovych P. (2016). Atomic intercalation to measure adhesion of graphene on graphite. Nat. Commun..

[46] Larciprete R., Colonna S., Ronci F., Flammini R., Lacovig P., Apostol N., Politano A., Feulner P., Menzel D., Lizzit S. (2016). Self-Assembly of Graphene Nanoblisters Sealed to a Bare Metal Surface. Nano Lett..

[47] Villarreal R., Lin P.-C., Faraji F., Hassani N., Bana H., Zarkua Z., Nair M.N., Tsai H.-C., Auge M., Junge F. (2021). Breakdown of Universal Scaling for Nanometer-Sized Bubbles in Graphene. Nano Lett..

[48] Blundo E., Surrente A., Spirito D., Pettinari G., Yildirim T., Chavarin C.A., Baldassarre L., Felici M., Polimeni A. (2022). Vibrational Properties in Highly Strained Hexagonal Boron Nitride Bubbles. Nano Lett..

[49] Blundo E., Felici M., Yildirim T., Pettinari G., Tedeschi D., Miriametro A., Liu B., Ma W., Lu Y., Polimeni A. (2020). Evidence of the direct-to-indirect band gap transition in strained two-dimensional WS_2_, MoS_2_, and WSe_2_. Phys. Rev. Res..

[50] Blundo E., Di Giorgio C., Pettinari G., Yildirim T., Felici M., Lu Y., Bobba F., Polimeni A. (2020). Engineered Creation of Periodic Giant, Nonuniform Strains in MoS_2_ Monolayers. Adv. Mater. Interfaces.

[51] Di Giorgio C., Blundo E., Pettinari G., Felici M., Lu Y., Cucolo A.M., Polimeni A., Bobba F. (2020). Nanoscale Measurements of Elastic Properties and Hydrostatic Pressure in H_2_-Bulged MoS_2_Membranes. Adv. Mater. Interfaces.

[52] Jain A., Bharadwaj P., Heeg S., Parzefall M., Taniguchi T., Watanabe K., Novotny L. (2018). Minimizing residues and strain in 2D materials transferred from PDMS. Nanotechnology.

[53] Hou Y., Ren X., Fan J., Wang G., Dai Z., Jin C., Wang W., Zhu Y., Zhang S., Liu L. (2020). Preparation of Twisted Bilayer Graphene via the Wetting Transfer Method. ACS Appl. Mater. Interfaces.

[54] Sanchez D.A., Dai Z., Wang P., Cantu-Chavez A., Brennan C.J., Huang R., Lu N. (2018). Mechanics of spontaneously formed nanoblisters trapped by transferred 2D crystals. Proc. Natl. Acad. Sci. USA.

[55] Pizzocchero F., Gammelgaard L., Jessen B.S., Caridad J.M., Wang L., Hone J., Bøggild P., Booth T.J. (2016). The hot pick-up technique for batch assembly of van der Waals heterostructures. Nat. Commun..

[56] Purdie D.G., Pugno N.M., Taniguchi T., Watanabe K., Ferrari A.C., Lombardo A. (2018). Cleaning interfaces in layered materials heterostructures. Nat. Commun..

[57] Haigh S.J., Gholinia A., Jalil R., Romani S., Britnell L., Elias D.C., Novoselov K.S., Ponomarenko L.A., Geim A.K., Gorbachev R. (2012). Cross-sectional imaging of individual layers and buried interfaces of graphene-based heterostructures and superlattices. Nat. Mater..

[58] Gasparutti I., Song S.H., Neumann M., Wei X., Watanabe K., Taniguchi T., Lee Y.H. (2020). How Clean Is Clean? Recipes for van der Waals Heterostructure Cleanliness Assessment. ACS Appl. Mater. Interfaces.

[59] Hou Y., Dai Z., Zhang S., Feng S., Wang G., Liu L., Xu Z., Li Q., Zhang Z. (2021). Elastocapillary cleaning of twisted bilayer graphene interfaces. Nat. Commun..

[60] Khestanova E., Guinea F., Fumagalli L., Geim A.K., Grigorieva I.V. (2016). Universal shape and pressure inside bubbles appearing in van der Waals heterostructures. Nat. Commun..

[61] Rao Y., Kim E., Dai Z., He J., Li Y., Lu N. (2023). Size-dependent shape characteristics of 2D crystal blisters. J. Mech. Phys. Solids.

[62] Dong W., Dai Z., Liu L., Zhang Z. (2023). Toward Clean 2D Materials and Devices: Recent Progress in Transfer and Cleaning Methods. Adv. Mater..

[63] Kretinin A.V., Cao Y., Tu J.S., Yu G.L., Jalil R., Novoselov K.S., Haigh S.J., Gholinia A., Mishchenko A., Lozada M. (2014). Electronic Properties of Graphene Encapsulated with Different Two-Dimensional Atomic Crystals. Nano Lett..

[64] Xu K., Cao P., Heath J.R. (2010). Graphene Visualizes the First Water Adlayers on Mica at Ambient Conditions. Science.

[65] Wu S., He F., Xie G., Bian Z., Ren Y., Liu X., Yang H., Guo D., Zhang L., Wen S. (2020). Super-Slippery Degraded Black Phosphorus/Silicon Dioxide Interface. ACS Appl. Mater. Interfaces.

[66] Wood J.D., Wells S.A., Jariwala D., Chen K.-S., Cho E., Sangwan V.K., Liu X., Lauhon L.J., Marks T.J., Hersam M.C. (2014). Effective Passivation of Exfoliated Black Phosphorus Transistors against Ambient Degradation. Nano Lett..

[67] Budania P., Baine P., Montgomery J., McGeough C., Cafolla T., Modreanu M., McNeill D., Mitchell N., Hughes G., Hurley P. (2017). Long-term stability of mechanically exfoliated MoS2 flakes. MRS Commun..

[68] Mirabelli G., McGeough C., Schmidt M., McCarthy E.K., Monaghan S., Povey I.M., McCarthy M., Gity F., Nagle R., Hughes G. (2016). Air sensitivity of MoS2, MoSe2, MoTe2, HfS2, and HfSe2. J. Appl. Phys..

[69] Kim H.H., Yang J.W., Jo S.B., Kang B., Lee S.K., Bong H., Lee G., Kim K.S., Cho K. (2013). Substrate-Induced Solvent Intercalation for Stable Graphene Doping. ACS Nano.

[70] Li Y., Wang B., Li W., Xu K. (2022). Dynamic, Spontaneous Blistering of Substrate-Supported Graphene in Acidic Solutions. ACS Nano.

[71] Zong Z., Chen C.-L., Dokmeci M.R., Wan K.-T. (2010). Direct measurement of graphene adhesion on silicon surface by intercalation of nanoparticles. J. Appl. Phys..

[72] Gao X., Yu X., Li B., Fan S., Li C. (2017). Measuring Graphene Adhesion on Silicon Substrate by Single and Dual Nanoparticle-Loaded Blister. Adv. Mater. Interfaces.

[73] Dai Z., Hou Y., Sanchez D.A., Wang G., Brennan C.J., Zhang Z., Liu L., Lu N. (2018). Interface-Governed Deformation of Nanobubbles and Nanotents Formed by Two-Dimensional Materials. Phys. Rev. Lett..

[74] Wang C., Shokuhfar T., Klie R.F. (2016). Precise In Situ Modulation of Local Liquid Chemistry via Electron Irradiation in Nanoreactors Based on Graphene Liquid Cells. Adv. Mater..

[75] Binder J., Dabrowska A.K., Tokarczyk M., Ludwiczak K., Bozek R., Kowalski G., Stepniewski R., Wysmolek A. (2023). Epitaxial Hexagonal Boron Nitride for Hydrogen Generation by Radiolysis of Interfacial Water. Nano Lett..

[76] Stolyarova E., Stolyarov D., Bolotin K., Ryu S., Liu L., Rim K.T., Klima M., Hybertsen M., Pogorelsky I., Pavlishin I. (2009). Observation of Graphene Bubbles and Effective Mass Transport under Graphene Films. Nano Lett..

[77] An H., Tan B.H., Moo J.G.S., Liu S., Pumera M., Ohl C.-D. (2017). Graphene Nanobubbles Produced by Water Splitting. Nano Lett..

[78] Jia P., Chen W., Qiao J., Zhang M., Zheng X., Xue Z., Liang R., Tian C., He L., Di Z. (2019). Programmable graphene nanobubbles with three-fold symmetric pseudo-magnetic fields. Nat. Commun..

[79] Lee J.H., Tan J.Y., Toh C.-T., Koenig S.P., Fedorov V.E., Neto A.H.C., Özyilmaz B. (2014). Nanometer Thick Elastic Graphene Engine. Nano Lett..

[80] Zhang X., Zhang H., Cao S., Zhang N., Jin B., Zong Z., Li Z., Chen X. (2020). Construction of Position-Controllable Graphene Bubbles in Liquid Nitrogen with Assistance of Low-Power Laser. ACS Appl. Mater. Interfaces.

[81] Bampoulis P., Teernstra V.J., Lohse D., Zandvliet H.J.W., Poelsema B. (2016). Hydrophobic Ice Confined between Graphene and MoS_2_. J. Phys. Chem. C.

[82] Pandey M., Kumar R. (2022). Polymer curing assisted formation of optically visible sub-micron blisters of multilayer graphene for local strain engineering. J. Phys. Condens. Matter.

[83] Yu C., Dai Z. (2023). Characterizing the wetting behavior of 2D materials: A review. J. Mater. Informatics.

[84] Vasu K.S., Prestat E., Abraham J., Dix J., Kashtiban R.J., Beheshtian J., Sloan J., Carbone P., Neek-Amal M., Haigh S.J. (2016). Van der Waals pressure and its effect on trapped interlayer molecules. Nat. Commun..

[85] Bunch J.S., Verbridge S.S., Alden J.S., Van Der Zande A.M., Parpia J.M., Craighead H.G., McEuen P.L. (2008). Impermeable atomic membranes from graphene sheets. Nano Lett..

[86] Koenig S.P., Boddeti N.G., Dunn M.L., Bunch J.S. (2011). Ultrastrong adhesion of graphene membranes. Nat. Nanotechnol..

[87] Yin P., Ma M. (2018). Efficient and Robust Fabrication of Microscale Graphene Drums. ACS Appl. Nano Mater..

[88] Wang G., Dai Z., Wang Y., Tan P., Liu L., Xu Z., Wei Y., Huang R., Zhang Z. (2017). Measuring Interlayer Shear Stress in Bilayer Graphene. Phys. Rev. Lett..

[89] Lloyd D.A.-O., Liu X., Boddeti N., Cantley L., Long R., Dunn M.L., Bunch J.S. (2017). Adhesion, Stiffness, and Instability in Atomically Thin MoS(2) Bubbles. Nano Lett..

[90] Sun P.Z., Yang Q., Kuang W.J., Stebunov Y.V., Xiong W.Q., Yu J., Nair R.R., Katsnelson M.I., Yuan S.J., Grigorieva I.V. (2020). Limits on gas impermeability of graphene. Nature.

[91] Sun P.Z., Yagmurcukardes M., Zhang R., Kuang W.J., Lozada-Hidalgo M., Liu B.L., Cheng H.-M., Wang F.C., Peeters F.M., Grigorieva I.V. (2021). Exponentially selective molecular sieving through angstrom pores. Nat. Commun..

[92] Nicholl R.J., Conley H.J., Lavrik N.V., Vlassiouk I., Puzyrev Y.S., Sreenivas V.P., Pantelides S.T., Bolotin K.I. (2015). The effect of intrinsic crumpling on the mechanics of free-standing graphene. Nat. Commun..

[93] Jimenez V.O., Kalappattil V., Eggers T., Bonilla M., Kolekar S., Huy P.T., Batzill M., Phan M.-H. (2020). A magnetic sensor using a 2D van der Waals ferromagnetic material. Sci. Rep..

[94] Jiang H., Zheng L., Liu Z., Wang X. (2020). Two-dimensional materials: From mechanical properties to flexible mechanical sensors. InfoMat.

[95] Lemme M.C., Wagner S., Lee K., Fan X., Verbiest G.J., Wittmann S., Lukas S., Dolleman R.J., Niklaus F., van der Zant H.S.J. (2020). Nanoelectromechanical Sensors Based on Suspended 2D Materials. Research.

[96] Khan Z.H., Kermany A.R., Öchsner A., Iacopi F. (2017). Mechanical and electromechanical properties of graphene and their potential application in MEMS. J. Phys. D Appl. Phys..

[97] Fonash S.J. (1990). An Overview of Dry Etching Damage and Contamination Effects. J. Electrochem. Soc..

[98] Saga K., Hattori T. (1996). Identification and Removal of Trace Organic Contamination on Silicon Wafers Stared in Plastic Boxes. J. Electrochem. Soc..

[99] Lee C., Kim H.W., Kim S. (2007). Organic contaminants removal by oxygen ECR plasma. Appl. Surf. Sci..

[100] Boddeti N.G., Koenig S.P., Long R., Xiao J., Bunch J.S., Dunn M.L. (2013). Mechanics of Adhered, Pressurized Graphene Blisters. J. Appl. Mech..

[101] Boddeti N.G., Liu X., Long R., Xiao J., Bunch J.S., Dunn M.L. (2013). Graphene blisters with switchable shapes controlled by pressure and adhesion. Nano Lett..

[102] Darlington T.P., Krayev A., Venkatesh V., Saxena R., Kysar J.W., Borys N.J., Jariwala D., Schuck P.J. (2020). Facile and quantitative estimation of strain in nanobubbles with arbitrary symmetry in 2D semiconductors verified using hyperspectral nano-optical imaging. J. Chem. Phys..

[103] Guinea F., Katsnelson M.I., Geim A.K. (2010). Energy gaps and a zero-field quantum Hall effect in graphene by strain engineering. Nat. Phys..

[104] Mansfield E.H. (1989). The Bending and Stretching of Plates.

[105] Dai Z., Rao Y., Lu N. (2022). Two-dimensional crystals on adhesive substrates subjected to uniform transverse pressure. Int. J. Solids Struct..

[106] Qi Z., Kitt A.L., Park H.S., Pereira V.M., Campbell D.K., Neto A.H.C. (2014). Pseudomagnetic fields in graphene nanobubbles of constrained geometry: A molecular dynamics study. Phys. Rev. B.

[107] Dai Z. (2024). Analytical Solutions for Circular Elastic Membranes Under Pressure. J. Appl. Mech..

